# Characterization and Comparative Profiling of miRNAs in Invasive *Bemisia tabaci* (Gennadius) B and Q

**DOI:** 10.1371/journal.pone.0059884

**Published:** 2013-03-20

**Authors:** Qiang Guo, Yun-Li Tao, Dong Chu

**Affiliations:** Key Lab of Integrated Crop Pest Management of Shandong Province, College of Agronomy and Plant Protection, Qingdao Agricultural University, Qingdao, Shandong Province, China; Kansas State University, United States of America

## Abstract

**Background:**

MicroRNAs (miRNAs) are small, conserved, non-coding RNAs that post-transcriptionally regulate gene expression. *Bemisia tabaci* (Gennadius) B and Q are two invasive and dominant whiteflies, and *B. tabaci* Q has been displacing B in China. Differences in biological traits (fecundity, host range, resistance to insecticides, etc.) as affected by miRNAs might be involved in the displacement. In this study, we performed high-throughput sequencing to identify miRNAs in *B. tabaci* B and Q.

**Results:**

We identified 170 conserved miRNAs and 15 novel candidates, and found significant differences in the expression of miRNAs between *B. tabaci* B and Q.

**Conclusion:**

Expression levels of miRNAs differ in *B. tabaci* B vs. Q. Additional research is needed to determine whether these differences are related to differences in the biology of *B. tabaci* B and Q, and whether these differences help explain why *B. tabaci* Q is displacing B in China.

## Introduction

MicroRNAs (miRNAs), which were first discovered in *C. elegans* [1], constitute a novel class of non-coding RNA species in viruses, plants, and animals [2]. By post-transcriptional regulation of gene expression, miRNAs play crucial roles in development [3]–[7], reproduction [8], stress response [9], [10], and in many other important molecular mechanisms and cellular processes [11]–[13].

In insects, repertoires of miRNAs have been mainly established for those species whose whole genomes have been sequenced. These include 12 *Drosophila* species [14], four hymenopterans (*Apis mellifera*, *Nasonia giraulti, N. longicornis*, and *N. vitripennis*) [15]–[17], three mosquitoes (*Aedes aegypti*, *Anopheles gambiae*, and *Culex quinquefasciatus*) [18]–[20], the pea aphid (*Acyrthosiphon pisum*) [21], the silkworm (*Bombyx mori*) [4], [6], [22]–[27], the butterfly (*Heliconius melpomene*) [28], the migratory locust (*Locusta migratoria*) [29], and the flour beetle (*Tribolium castaneum*) [30], [31].

*Bemisia tabaci* (Gennadius) (Hemiptera: Aleyrodidae) is an important agricultural pest worldwide that attacks more than 600 plant species including food, fiber, and ornamental plants under field and greenhouse conditions [32], [33]. By phloem feeding, contaminating leaves and fruits with honeydew, and transmitting more than 110 kinds of plant viruses, adult and immature instars of *B. tabaci* cause billions of dollars of annual loss worldwide [34], [35]. In particular, *B. tabaci* often causes outbreaks of plant-pathogenic viruses [36], [37].

*Bemisia tabaci* is currently regarded as a cryptic species complex that contains at least 24 morphologically indistinguishable species [38]. Two members of this complex, Middle East-Asia Minor 1 (commonly known as *B. tabaci* biotype B, herein referred to as *B. tabaci* B) and Mediterranean (commonly known as *B. tabaci* biotype Q, herein referred to as *B. tabaci* Q), have now spread well beyond their home ranges as a consequence of trade in ornamental plants [38]. In China, *B. tabaci* B and Q are the main whiteflies in agricultural areas and have caused severe damage to many crops [39]–[41]. We previously reported that the ratio of abundances of *B. tabaci* Q to *B. tabaci* B has been increasing and that Q is displacing B on cotton, eggplant, and other plants in Shandong Province of China [42], [43]. Teng *et al.* [40] also found that *B. tabaci* Q has become dominant across China and suggested that *B. tabaci* Q has been displacing non-Q whiteflies in many regions such as Shanxi, Henan, Hubei, Jiangsu, Zhejiang, Hunan, and Hainan provinces.

As noted earlier, many biological differences between *B. tabaci* B and Q have been documented. *B. tabaci* Q had significantly greater reproductive parameters than B in winter weeds [44], had shorter developmental times than B on sweet pepper at constant temperatures [45], had better survival than B under low as well as high temperature conditions [46], and had greater resistance to neonicotinoides and pyriproxyfen insecticides than B [47]–[49]. All of these would seem to at least partially explain the displacement of B by Q [50]. The genetic differences between B and Q have also been studied recently, and several genes involved in metabolism and insecticide resistance were considered as possibly contributing to the divergence of the two whitefly species [51]. Because miRNAs are now recognized as critical regulators of gene expression and animal development, the identification and comparison of miRNAs in *B. tabaci* B and Q could provide new information about the biological differences in these biotypes. The differences between miRNAs of B and Q might also explain the biological differences because miRNAs play an important role in a wide range of cellular and developmental process including cell proliferation [52], cell differentiation [53], [54], the cell cycle [55], [56], metabolism [57], developmental timing [58], reproduction [8], apoptosis [59], and others [11].

In recent years, high-throughput sequencing technology has been widely used to analyze the characteristics of miRNA within the organisms [60]–[62]. For example, by high-throughput sequencing of miRNA, Marco *et al*. [60] characterized 203 miRNAs from the red flour beetle *Tribolium castaneum*; Liu *et al*. [61] analyzed areas of skin where the cashmere grows in anagen and found that the miRNAs that were coexpressed in goat and sheep were located in the same region of the respective chromosomes and may play an essential role in skin and follicle development; Shao *et al*. [62] analyzed Arabidopsis (*Arabidopsis thaliana*) and rice (*Oryza sativa*) and found that the accumulation levels of several miRNA*s could be much higher than those of their miRNA partners in certain organs, mutants and/or AGO-associated silencing complexes of both Arabidopsis and rice.

In this study, we performed high-throughput sequencing to identify miRNAs in *B. tabaci* B and Q, and identified 170 conserved miRNAs and 15 novel candidates. We compared the expression of miRNAs in B and Q to identify differentially expressed miRNAs. The results indicate significant differences in the expression of miRNAs between *B. tabaci* B and Q.

## Materials and Methods

### Whitefly Colony and RNA Extraction

*Bemisia tabaci* B and Q colonies were reared on cotton leaves in growth chambers at 26±1°C and with a 16/8 h light/dark photoperiod. Adult whiteflies (*B. tabaci* B and Q) were collected and homogenized in Trizol agent RNAiso Plus (TaKaRa, Dalian, China). Total RNA was extracted from B and Q according to the manufacturer’s instructions and was quantified with an Agilent 2100 Bioanalyzer.

### Confirmation of *B. tabaci* B and Q

The identities of *B. tabaci* B and Q were confirmed based on the cleaved amplified polymorphic sequences (CAPS) of *mtCOI* (mitochondrial cytochrome oxidase I) with the restriction endonucleases *Vsp*I and *Stu*I [43], [63]. Genomic DNA was extracted from individual adult whiteflies according to the lysis method of Frohlich *et al.* [64]. The *mtCOI* fragments were amplified using primers C1-J-2195 (5′-TTGATTTTTTGGTCATCCAGAAGT-3′) and R-BQ-2819 (5′- CTGAATATCGRCGAGGCATTCC -3′) [63]. The 20 µL PCR reaction mixture contained 2 µL of 10×reaction buffer supplemented with 1.5 mM MgCl_2_, 0.2 µM of each primer, 0.2 mM of each dNTP, 1 unit of *Taq* DNA polymerase, and 2 µL of each template cDNA. Cycling conditions were as follows: 5 min at 94°C; 35 cycles of 1 min at 94°C, 1 min at 52°C, and 1 min at 72°C; and finally 10 min at 72°C. PCR products were electrophoresed and visualized by ethidium bromide staining. The *mtCOI* fragment (approximately 620 bp) was first cleaved by *Vsp*I [65], and then the uncut fragment was cleaved by *Stu*I [66]. Specimens whose *mtCOI* fragments were cut by *Vsp*I were identified as *B. tabaci* Q, whereas specimens whose *mtCOI* fragments were cut by *Stu*I were identified as *B. tabaci* B [43], [63].

### Small RNA Library Preparation and High-throughput Sequencing

For HiSeq deep sequencing, the small RNA samples were prepared as described previously [29]. In brief, RNA fragments with fewer than 40 nt were isolated from total RNA on a 15% Novex TBE-urea PAGE gel. Then, a 5′ adaptor (Illumina, San Diego, CA, USA) was ligated to the purified small RNAs, and the ligation products were purified on a 15% Novex TBE-urea PAGE gel. The 5′ ligation products were then ligated to a 3′ adaptor (Illumina), and products with 5′ and 3′ adaptors were size-fractionated on a 10% Novex TBE-urea PAGE gel. Subsequently, small RNAs ligated with adaptors were reverse transcribed and then subjected to PCR amplification. The amplification products were purified on a 6% Novex TBE PAGE gel. The purified DNA fragments were used for clustering and sequencing by HiSeq high-throughput sequencing technology at the Beijing Genomics Institute, Shenzhen.

### Discovery of Conserved miRNAs

The tags under 40 nt sequence from HiSeq sequencing were first subjected to data cleaning, which included removal of the low quality tags and several kinds of contaminants. The distribution of the lengths of the clean tags was then summarized, and the clean tags were assigned to two groups including the summary of unique tags and total tags. The clean tags were annotated into different categories to discard rRNAs, tRNAs, snRNAs, and snoRNAs using Rfam database (version 10.1). Because there was no information concerning miRNAs of *B. tabaci* in the miRBase v17.0, the remaining small RNA tags were aligned to the miRNA precursors/mature miRNAs of all animals in the miRBase v17.0 [67], [68]. Sequences in our libraries that were identical to or related to (having four or fewer nucleotide substitutions) sequences from *Drosophila melanogaster* or other insects (*A. aegypti*, *A. mellifera*, *B. mori*, and *T. castaneum*) were identified as conserved miRNAs.

### Prediction of Novel miRNA Candidates

The characteristic hairpin structure of miRNA precursors can be used to predict novel miRNA candidates. Because there were no completed genome sequences, 27,288 nucleotide sequences of *Bemisia tabaci* obtained from NCBI (published by Zhejiang University) were used as a reference for novel miRNA prediction. The prediction software Mireap was used to predict novel miRNA candidates by exploring the secondary structure, the Dicer cleavage site, and the minimum free energy of the unannotated small RNA tags that could be mapped to the genome. The rules used to identify novel miRNA candidates were based on those suggested by Allen *et al.* [69] and Schwab *et al.* [70]: (1) novel miRNAs should have no more than four mismatches between sRNA & target (G-U bases count as 0.5 mismatches); (2) novel miRNAs should have no more than two adjacent mismatches in the miRNA/target duplex; (3) novel miRNAs should have no adjacent mismatches in positions 2–12 of the miRNA/target duplex (5′ of miRNA); (4) novel miRNAs should have no mismatches in positions 10–11 of the miRNA/target duplex; (5) novel miRNAs should have no more than 2.5 mismatches in positions 1–12 of the miRNA/target duplex (5′ of miRNA); (6) minimum free energy (MFE) of the miRNA/target duplex should be ≥75% of the MFE of the miRNA bound to its perfect complement.

### Comparing the Expression of miRNAs between *B. tabaci* B and Q

We compared the expression of miRNAs between *B. tabaci* B and Q to identify differentially expressed miRNAs. The expression of miRNAs in the two libraries was visualized on a scatter plot in which expression of B miRNAs was plotted against expression of Q miRNAs after expression levels were normalized and then transformed into fold-change values (see below). The threshold of a fold change more than 2 was considered significant difference. The procedure had two parts. First, the expression of miRNA in the two libraries (Q as control and B as treatment) was normalized to transcripts per million (TPM). If the normalized expression of a miRNA was zero, it was modified to 0.01 to enable calculation. If the normalized expression of a miRNA was less than 1 in both B and Q libraries, it was ignored to compare for low expression. The normalization formula was:

Normalized expression = Actual miRNA count/Total count of clean reads*1000000. Second, the normalized data were used to calculate fold-change values and *P*-values, and a scatter plot of the fold-change values was generated. Fold-change was calculated as: Fold-change = log_2_(B/Q). The *P*-value was calculated as
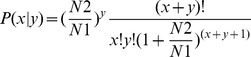
x represents Q; y represents B; N1 represents the normalized expression of a miRNA in Q library; N2 represents the normalized expression of the same miRNA in B library.

## Results

### *B. tabaci* has a Complex Population of Small RNAs

**Figure 1:**
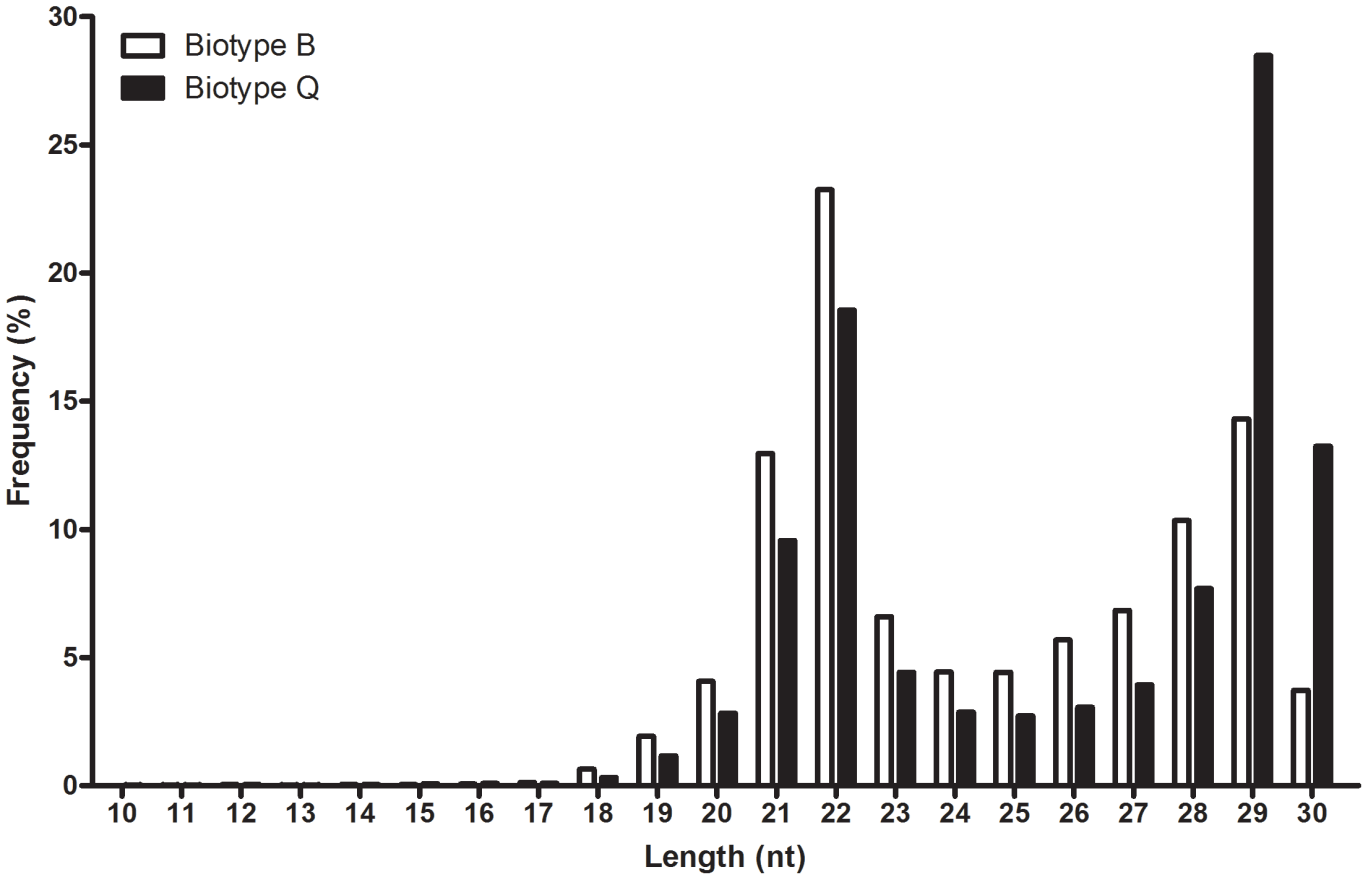
Frequency distribution of miRNA sequence lengths of *B. tabaci* B and Q.

HiSeq high-throughput sequencing technology was used to identify miRNAs in *B. tabaci* B and Q. Two libraries of small RNAs were constructed, one from B and the other from Q. We obtained 17,953,732 reads from the B library, and 16,448,832 reads from the Q library. Low-quality sequences and those shorter than 18 nt were removed, leaving 17,451,513 reads (2,871,240 unique sequences) in the B library and 15,977,474 reads (3,041,144 unique sequences) in the Q library. The distribution of sequence lengths indicated that both libraries were enriched with small RNAs of 21–23 nt (42.8% and 32.6% of all reads in B and Q libraries, respectively) (Fig. 1), which is considered the standard size of miRNAs. Another type of RNA sequence found in both libraries was 28–30 nt long, corresponding to pi-RNA-like sequences, and represented 28.4% and 49.4% of the reads in B and Q libraries, respectively. In these libraries, sequence length was limited to a maximum of 30 nt.

**Figure 2:**
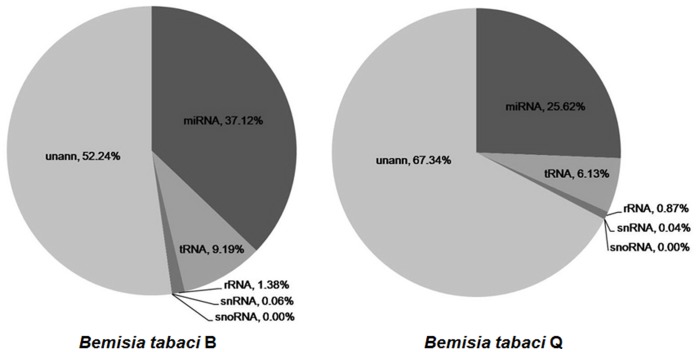
Composition of the small RNA libraries in *B. tabaci* B and Q.

Subsequent sequence analysis eliminated reads corresponding to rRNAs, tRNAs, snRNAs, and snoRNAs. Another two large fractions of reads were derived from unannotated genome sites (52.2% and 67.3% of high-quality clean reads in B and Q libraries) and miRNAs (37.1% and 25.6%, respectively) (Fig. 2). After successive filtering of these data sets, we identified 52,977 unique miRNA genes comprising 1,504 miRNAs in the B library, and 39,266 unique miRNA genes comprising 1,182 miRNAs in the Q library. Although some miRNAs were very abundant in these data sets, most miRNAs were sequenced only a few times, indicating that the sampled *B. tabaci* might have a large and complex miRNA population. For example, 37,186 of 52,977 unique miRNA genes were sequenced only one time in the B library. The unique data set with read counts was used to identify conserved and novel miRNAs in *B. tabaci*.

### Identification of Conserved miRNAs

To identify conserved miRNAs in *B. tabaci*, we searched all small RNA sequences against the currently miRNAs of all animals in miRBase v17.0 using BLASTn [67], [68]. In total, 1,504 miRNAs were found in the B library, and 1,182 miRNAs were found in the Q library. Sequences in our libraries that were identical to or related to (having four or fewer nucleotide substitutions) sequences from *D. melanogaster* or other insects (*A. aegypti*, *A. mellifera*, *B. mori*, and *T. castaneum*) were identified as conserved miRNAs. After BLASTn searches and further sequence analysis, a total of 170 conserved miRNAs identified from the miRNAs were found in both B and Q libraries (Table 1). In these conserved miRNAs, there were nine miRNA families containing five or more miRNAs (Table 1). The identified miRNA families are conserved in a variety of animal species. For example, let-7 [71], miR-9 [72], [73], miR-10 [74], miR-133 [75], [76], and miR-263 [6] have been found in 100, 63, 46, 89, and 74 animal species, respectively.

**Table 1:** Conserved miRNAs from *B. tabaci*.

miR-name	Sequence (5′-3′)	Length (nt)	Conserved in other insects					Reads		Normalrized reads	
			aae	ame	bmo	dme	tca	B	Q	B	Q
bantam	UGAGAUCAUCGUGAAAGCUGAU	22	+	+	+	+	+	449558	319046	25760.4	19968.4881
bantam*	UGAGAUCAUCAUGAAAGCUGA	21	+	+	+	+	+	202	149	11.5749	9.3256
bantam-3p	UGAGAUCAUCAUGAAAGCUGAU	22	+	+	+	+	+	183	142	10.4862	8.8875
bantam-b	UGAGAUCAUCGCGAAAGCUGAU	22	+	+	+	+	+	270	175	15.4714	10.9529
let-7	UGAGGUAGUAGGUUGUAUAGUA	22	+	+	+	+	+	210891	112112	12084.4	7016.8789
let-7-5p	UGAGGUAGUAGGUUGUAUAGU	21	+	++	++	++	+	224460	119274	12861.92	7465.135
let-7e	UGAGGUAGUAGGUUGUUUAGU	21	+	+	+	+	+	69	41	3.9538	2.5661
let-7f	UGAGGUAGUAGAUUGUAUAGU	21	+	+	+	+	+	31	13	1.7764	0.8136
let-7g	UGAGGUAGUAGUUUGUAUAG	20	+	+	+	+	+	23	10	1.3179	0.6259
miR-1-3p	UGGAAUGUAAAGAAGUAUGGAG	22	++	++	++	++	++	1832382	970193	104998.5	60722.5523
miR-1a	UGGAAUGUAAAGAAGUAUGGAUAU	24	+	+	+	+	+	336840	237983	19301.48	14894.9077
miR-1b	UGGAAUGUAAAGAAGUAUGGUU	22	+	+	+	+	+	46036	35801	2637.938	2240.7171
miR-1c	UGGAAUGUAAAGAAGUAUGAGA	22	+	+	+	+	+	341	4775	19.5399	298.8583
miR-2a	UAUCACAGCCAGCUUUGAUG	20	++	++	++	++	++	1768	1684	101.3093	105.3984
miR-2a-1-5p	UCAUCAAAAUGGUUGUGGAAUG	22				+		1085	833	62.1723	52.1359
miR-2a-3p	UCACAGCCAGCUUUGAUGAG	20	++	++	++	++	++	5133	2784	294.1292	174.2453
miR-2b	UAUCACAGCCACUUUGAUGAACU	23		+	+	+	+	5370	2521	307.7097	157.7846
miR-2b-2-5p	UCGUCAAAUGGUUGUGAAGUG	21				+	+	1369	580	78.4459	36.3011
miR-2c	UCACAGCCAGCUUUGAUGAGU	21	+	+	+	+	+	2131	1192	122.1098	74.605
miR-7	UGGAAGACUAGUGAUUUUGUU	21	++	++	++	++	++	9070	6040	519.7257	378.0322
miR-7-5p	UGGAAGACUAGUGAUUUUGUUGU	23	++	++	++	++	++	8511	5643	487.6941	353.1847
miR-8	UAAUACUGUCAGGUAAUGAUG	21	+	+	+	+	+	106163	68555	6083.312	4290.728
miR-8-3p	UAAUACUGUCAGGUAAAGAUGUC	23	++	++	++	++	++	9	93	0.5157	5.8207
miR-8a	UAAUACUGUCAGGUAACGAUG	21	+	+	+	+	+	24	16	1.3752	1.0014
miR-9	UCUUUGGUUACCUAGCUGUGUG	22	+	+	+	+	+	4	21	0.2292	1.3144
miR-9a	UCUUUGGUAUUCUAGCUGUAGGAU	24	+	+	+	+		105346	70800	6036.497	4431.2386
miR-9b	UCUUUGGUUACCUAGCUGUAUG	22	+	+	+	+	+	9786	10071	560.7537	630.3249
miR-9b-3p	AUAAAGCUGGAUUACCAAAGCG	22	+	+	+	+	+	73	61	4.183	3.8179
miR-9b-5p	UCUUUGGUAUUUUAGCUGUAG	21	+	+	+	+	+	351	180	20.1129	11.2659
miR-9c-5p	UCUUUGGUAUUCUAGCUGUAGG	22	+	+	+	+	+	109953	73652	6300.485	4609.7399
miR-9d-3p	AUAAAGCUGGAUUACCAAAUCG	22	+	+	+	+	+	95	48	5.4437	3.0042
miR-10	ACCCUGUAGAUCCGAAAUUUGU	22	+	+	+	+	+	25070	11485	1436.552	718.8245
miR-10*	AAAUUCGGUUCUAGAGAGGUU	21			++	++	++	4349	2325	249.2048	145.5174
miR-10-3p	CAAAUUCGGUUCUAGAGAGGU	21			++	++	++	4489	2400	257.227	150.2115
miR-10a	UACCCUGUAGAUCCGAAAUUUG	22	+	+	+	+	+	24759	11331	1418.731	709.1859
miR-10c	ACCCUGUAGAUCGAAAUUUGU	21	+	+	+	+	+	1	103	0.0573	6.4466
miR-12	UGAGUAUUACAUCAGAUAUUUGA	23	+	+		+		12268	7732	702.9763	483.9313
miR-12-5p	UGAGUAUUACAUCAGAUAUUUG	22	+	+	+	+	+	12539	7870	718.505	492.5685
miR-13a-3p	UAUCACAGCCACUUUGAUGAAC	22	+	+	+	+	+	5276	2469	302.3234	154.5301
miR-13a-5p	UCGUCAAAUGGUUGUGAAGU	20				+	+	1338	568	76.6696	35.5501
miR-13b	UAUCACAGCCAUUUUUGACGUG	22		+	++			1905	1243	109.1596	77.797
miR-14	UCAGUCUUUUUCUCUCUCCUAU	22	++	+	+	+	+	6610	5453	378.7637	341.293
miR-14-3p	UCAGUCUUUUUCUCUCUCCUA	21	++	++	++	++	++	6241	5223	357.6194	326.8977
miR-29a	UAGCACCAUUUGAAAUCAGA	20	+	+	+	+	+	33	31	1.891	1.9402
miR-29b	UAGCACCAUUUGAAAUCAGUG	21	+	+	+	+	+	1267	720	72.6012	45.0634
miR-31	AGGCAAGAUGUUGGCAUAGCUG	22	+	+	+	+	+	1825	1042	104.5755	65.2168
miR-34	UGGCAGUGUGGUUAGCUGGUU	21	++	+	++	++	+	5596	4347	320.6599	272.0705
miR-34-5p	UGGCAGUGUGGUUAGCUGGUUGUG	24	+	+	+	++	+	5161	3942	295.7337	246.7224
miR-71	UGAAAGACAUGGGUAGUGAGAUG	23	+	+	+		+	2927	2052	167.7218	128.4308
miR-71*	UCUCACUACCUUGUCUUUCAU	21	++		++		++	965	747	55.2961	46.7533
miR-71-3p	UCUCACUACCUUGUCUUUCAUG	22	++		+		++	984	760	56.3848	47.567
miR-71c	UGAAAGACAUGGGUAGUGAGA	21	+	+	+		++	3127	2164	179.1822	135.4407
miR-72	AGGCAAGAUGUUGGCAUAGCUGA	23	+	+	+	+	+	1860	1054	106.581	65.9679
miR-79	AUAAAGCUGGAUUACCAAAGCGU	23	+	+	+	+	+	72	59	4.1257	3.6927
miR-80	UGAGAUCAUAGUGAAAGCUGA	21	+	+	+	+	+	107	72	6.1313	4.5063
miR-81	UGAGAUCAUCGUGAAAGCUGA	21	+	+	+	+	+	487501	349386	27934.6	21867.4116
miR-82	UGAGAUCAUCGUGAAAGCCGA	21	+	+	+	+	+	3155	2928	180.7866	183.258
miR-87	GUGAGCAAAGUUUCAGGUGUGU	22	+	+	+	+	+	2231	1414	127.8399	88.4996
miR-87a	GUGAGCAACGUAUCAGGUGUCU	22	+	+			+	3124	1982	179.0103	124.0496
miR-87a-3p	GUGAGCAAAGUUUCAGGUGUG	21	+	+	+	+	++	2203	1398	126.2355	87.4982
miR-87b-3p	GUGAGCAAAGUUUCAGGUGUGUU	23	+	+	+	+	+	2173	1376	124.5164	86.1212
miR-92a	UAUUGCACUUGUCCCGGCCUAU	22	+	+	+	+	+	2878	1345	164.9141	84.181
miR-92a-3p	AAUUGCACUAGUCCCGGCCUA	21	+	+	+	+	+	46	73	2.6359	4.5689
miR-92b	UAUUGCACUUGUCCCGGCCUU	21	+	+		+	+	225	140	12.8929	8.7623
miR-92b-3p	AAUUGCACUAGUCCCGGCCUGC	22	+	+	+	++	+	6483	3073	371.4864	192.3333
miR-92c	AUUGCACUAGUCCCGGCCUGCU	22	+	+	+	+	+	4976	2	285.1329	0.1252
miR-99a	AACCCGUAGAUCCGACCUUGU	21	+	+	+	+	+	48	34	2.7505	2.128
miR-100	AACCCGUAGAUCCGAACUUGU	21	++	++	++	+	++	126119	78502	7226.823	4913.2923
miR-100-5p	AACCCGUAGAUCCGAACUUGUGAA	24	+	+	+	+	+	115257	71338	6604.413	4464.911
miR-100b	AACCCGUAGAUUCGAACUUG	20	+	+	+	+	+	208	102	11.9187	6.384
miR-124-3p	UAAGGCACGCGGUGAAUGCCAUU	23	+	+	+	+	+	35	263	2.0056	16.4607
miR-125	CCCCUGAGACCCUAAUUUGUGA	22	+	+		+	+	69	4	3.9538	0.2504
miR-125b-5p	UCCCUGAGACCCUAAUUUGUGA	22	+	+		+	+	5232	2988	299.8021	187.0133
miR-133	UUGGUCCCCUUCAACCAGCUG	21	++	++	++	++	++	540	460	30.9429	28.7905
miR-133-3p	UUGGUCCCCUUCAACCAGCUGU	22	++	++	++	++	++	518	441	29.6822	27.6014
miR-133-5p	AGCUGGUUGAACCUGGGUCA	20				+	+	29	11	1.6617	0.6885
miR-133b	UUGGUCCCCUUCAACCAGCU	20	++	++	++	++	++	533	452	30.5418	28.2898
miR-133c	UUGGUCCCCUUCAACCAGCUGC	22	+	+	+	+	+	275	248	15.7579	15.5219
miR-137	UUAUUGCUUGAGAAUACACGU	21	+	++	++	+	++	11769	8984	674.3828	562.2916
miR-137-3p	UAUUGCUUGAGAAUACACGUAG	22	++	+	+	++	+	11917	9090	682.8634	568.926
miR-137b	UUAUUGCUUGAGAAUACACGUAG	23	+	+	+	+	+	11768	8972	674.3255	561.5406
miR-182	CUUGGCACUGGAAGAAUUCACU	22	+	+	+	+	+	50427	34216	2889.549	2141.515
miR-183	UAUGGCACUGGAAGAAUUCACG	22	+	+	+	+	+	744	9136	42.6324	571.805
miR-184	UGGACGGAGAACUGAUAAGGG	21	++	++	++	++	++	892323	648079	51131.56	40562.0438
miR-184-3p	UGGACGGAGAACUGAUAAGGGC	22	++	++	++	++	++	771607	569850	44214.33	35665.838
miR-184b	UGGACGGAGAACUGAUAAGGA	21	+	+	+	+	+	249618	152208	14303.52	9526.412
miR-190	AGAUAUGUUUGAUAUUCUUGGU	22	++	++	++	++	+	5445	3437	312.0073	215.1154
miR-190-5p	AGAUAUGUUUGAUAUUCUUGG	21	++	++	++	++	++	5577	3528	319.5711	220.8109
miR-200	UAAUACUGUCAGGUGAUGAUG	21	+	+	+	+	+	31	7	1.7764	0.4381
miR-200b	UAAUACUGUCAGGUAAUGAUGUU	23	+	+	+	+	+	103148	66907	5910.548	4187.5831
miR-200b*	AUCUUACUGGGCAGCAUUGGA	21	+		+	+	+	4051	1846	232.1289	115.5377
miR-200c	UAAUACUGCCAGGUAAUGAUG	21	+	+	+	+	+	53	30	3.037	1.8776
miR-206	UGGAAUGUAAGGAAGUAUGG	20	+	+	+	+	+	18289	5764	1047.989	360.7579
miR-210	UUGUGCGUGUGACAGCGGCU	20	+	++	++	++	++	1434	768	82.1705	48.0677
miR-210*	AGCUGCUGGACACUGCACAAGA	22			+	+	+	117	85	6.7043	5.32
miR-210-3p	UUGUGCGUGUGACAGCGGCUA	21	+	++	+	++	++	1323	691	75.81	43.2484
miR-210-5p	AGCUGCUGGACACUGCACAAGAUG	24				+	+	81	67	4.6414	4.1934
miR-228	AAUGGCACUAGAAGAAUUCACG	22	+	+	+	+	+	175249	128907	10042.05	8068.0463
miR-252	CUAAGUAGUAGCGCCGAAGGU	21			+	+	+	844	682	48.3626	42.6851
miR-252-5p	CUAAGUACUAGCGCCGCAGGAG	22	+	+	+	+	+	1	17	0.0573	1.064
miR-252a	CUAAGUACUCCGUGCCGCAGGA	22			+	+		6797	3528	389.4791	220.8109
miR-252b	CUAAGUAGUAGCGCCGAAGGUGA	23					+	746	587	42.747	36.7392
miR-252b-5p	UAAGUAGUAGCGCCGAAGGU	20	+	+	+	+	+	789	634	45.211	39.6809
miR-263	UGUGGCACUGGAAGAAUUCACG	22	+	+	+	+	+	148489	8	8508.661	0.5007
miR-263a	AAUGGCACUAGAAGAAUUCAC	21	+	+	+	+	+	149392	114108	8560.404	7141.8048
miR-263a-5p	AAUGGCACUAGAAGAAUUCACGGG	24	+	+	+	+	+	142136	108548	8144.623	6793.8148
miR-263b	CUUGGCACUGGAAGAAUUCACAGA	24	+	++	+	+	++	150352	96869	8615.414	6062.8482
miR-263b-5p	CUUGGCACUGGAAGAAUUCAC	21	+	++	++	++	++	152738	100503	8752.135	6290.2934
miR-275	UCAGGUACCUGAAGUAGCGCG	21	++	++	++	++	++	30183	19544	1729.535	1223.2221
miR-275-3p	UCAGGUACCUGAAGUAGCGCGC	22	+	++	++	++	++	28391	18513	1626.85	1158.6938
miR-276	UAGGAACUUCAUACCGUGCUC	21	++	++	++	++	++	295077	193301	16908.39	12098.3455
miR-276-5p	AGCGAGGUAUAGAGUUCCUACGU	23	+		+	+	++	93	72	5.3291	4.5063
miR-276a-3p	UAGGAACUUCAUACCGUGCUCU	22	+	++	++	++	++	300783	196581	17235.35	12303.6345
miR-276b-3p	UAGGAACUUAAUACCGUGCUCU	22	+	+	+	++	+	72	61	4.1257	3.8179
miR-277	UAAAUGCACUAUCUGGUACGAC	22	++	++	++	++	++	9223	6114	528.4929	382.6637
miR-277-3p	UAAAUGCACUAUCUGGUACGACA	23	+	++	++	++	++	8984	5932	514.7978	371.2727
miR-278	UCGGUGGGACUUUCAUCUGA	20	+	+		+		198454	127488	11371.74	7979.2338
miR-279	UGACUAGAUCCACAUUCAUCCA	22	+	+	+	+	+	3	24	0.1719	1.5021
miR-279b	UGACUAGAUUUUCACUCAUUCA	22	+	+	+	+	+	1778	881	101.8823	55.1401
miR-279d	UGACUAGAUUUUCACUCAUUC	21	+	+	+	+	+	1821	896	104.3463	56.079
miR-279e	UGACUAGAGAUACACUCGCU	20	+	+	+			396	74	22.6914	4.6315
miR-281	UGUCAUGGAGUUGCUCUCUUUG	22	+	++	+	+	+	510	407	29.2238	25.4734
miR-281-2-5p	AAGAGAGCUAUCCUUCGACAGU	22	+		+	+	+	242	226	13.867	14.1449
miR-281-3p	UGUCAUGGAGUUGCUCUCUUU	21	+	++	+	+	++	494	395	28.307	24.7223
miR-285	UAGCACCAUGGAAUUCAGCUUU	22			+	+	+	206	128	11.8041	8.0113
miR-285-3p	UAGCACCAUUUGAAAUCAGUGC	22	+	+	+	+	+	1225	693	70.1945	43.3736
miR-305	AUUGUACUUCAUCAGGUGCUCUGG	24	++	+	+	+	+	1917	1589	109.8472	99.4525
miR-305-5p	AUUGUACUUCAUCAGGUGCUCUG	23	++	++	++	++	+	1905	1529	109.1596	95.6972
miR-306	UCAGGUACUGAGUGACUCUGA	21	++	++	+	+		11424	7708	654.6137	482.4292
miR-306-5p	UCAGGUACUGAGUGACUCUAA	21	+	+	+	+		96	1	5.501	0.0626
miR-307	UCACAACCUCCUUGAGUGAGC	21	+	+	+	+	+	390	305	22.3476	19.0894
miR-307a-3p	UCACAACCUCCUUGAGUGAG	20	+	+	++	++	++	399	318	22.8633	19.903
miR-315	UUUUGAUUGUUGCUCAGAAAGCC	23	+	+		+	+	31934	20078	1829.87	1256.6442
miR-315-5p	UUUUGAUUGUUGCUCAGAAAGC	22	++	++		++	++	33300	20952	1908.144	1311.3462
miR-316	UGUCUUUUCCCGCUUUGCUGCC	22		+	+		+	1100	925	63.0318	57.894
miR-316*	UGUCUUUUCCCGCUUUGCUGU	21		+	+		+	652	353	37.3607	22.0936
miR-316-5p	UGUCUUUUCCCGCUUUGCUGC	21		+	+		+	1210	976	69.335	61.086
miR-317	UGAACACAGCUGGUGGUAUCU	21	++	++	+	+	++	244251	172084	13995.98	10770.4134
miR-317-3p	UGAACACAGCUGGUGGUAUCA	21	+	+	+	+	+	21219	15822	1215.883	990.2692
miR-375	UUUGUUCGUUCGGCUCGAGUUA	22	+	++		+	+	40466	21736	2318.767	1360.4153
miR-375-3p	CUUGUUCGUUCGGCUCGAGU	20	+	+		+	+	2	90	0.1146	5.6329
miR-750	CCAGAUCUAACUCUUCCAGCUCU	23		+				32168	20981	1843.279	1313.1613
miR-750-3p	UCAGAUCUAACUCUUCCAGCUCU	23		+				169	95	9.684	5.9459
miR-927*	CAAAGCGUUUGAAUUCUGGAAA	22	+			+	+	1960	1579	112.3112	98.8266
miR-927-3p	CAAAGCGUUUGAAUUCUGGAAC	22	+			+	+	16281	12197	932.9277	763.3873
miR-927-5p	UUUAGAAUGCUUACGCUUUACC	22		+	+	+	+	274	159	15.7006	9.9515
miR-929	AAAUUGACUCUAGUAGGGAG	20	+	+	+	++	+	2366	1336	135.5756	83.6177
miR-29-5p	AAAUUGACUCUAGUAGGGAGU	21	+	+	+	++	+	2412	1346	138.2115	84.2436
miR-965-3p	UAAGCGUAUAGCUUUUCCCCU	21	+		++	++	++	18	13	1.0314	0.8136
miR-971	UUGGUGUUCUACCUUACAGUG	21		++		+		172	94	9.8559	5.8833
miR-981	UUCGUUGUCGCCGAAAACUCG	21	+			+	+	2478	1235	141.9934	77.2963
miR-993*	CUACCCUGUAGAUCCGGGCUUUUG	24	+		+	+	+	2565	1533	146.9787	95.9476
miR-993-3p	GAAGCUCGUCUCUACAGGUAUC	22		++	++	++	++	1265	929	72.4866	58.1444
miR-993-5p	UACCCUGUAGAUCCGGGCUUUUG	23	+		+	+	+	2601	1547	149.0415	96.8238
miR-993a*	UACCCUGUAGAUCCGGGCUUUU	22	+		++	+	+	2592	1547	148.5258	96.8238
miR-993b*	UACCCUGUAGAUCCGGGCUUU	21	+		++	+	+	2596	1553	148.755	97.1993
miR-996	UGACUAGAGUUACACUCGUCA	21		+				3037	1833	174.025	114.724
miR-998	UAGCACCAUGGAAUUCAGCUU	21	+		+	+	+	672	387	38.5067	24.2216
miR-1000	AUAUUGUCCUGUCACAGCAGUA	22	+	+	+	+	+	1704	973	97.642	60.8982
miR-1000-5p	AUAUUGUCCUGUCACAGCAG	20	++	+	++	++	+	1771	1009	101.4812	63.1514
miR-1175-3p	UGAGAUUCAACUCCUCCAUCUU	22	+	+	+		+	425	198	24.3532	12.3924
miR-2765	UGGUAACUCCACCACCGUUGGC	22	++		++		++	2794	1710	160.1007	107.0257
miR-2779	UCAUAUCCGGCUCGGAGGACCA	22			+			433	195	24.8116	12.2047
miR-2796	GUAGGCCGGCGGAAACUACU	20		++	++		++	6233	2486	357.161	155.5941
miR-2796-3p	GUAGGCCGGCGGAAACUACUUGC	23		++	++		++	5496	2198	314.9297	137.5687
miR-3049	UCCGUCCAACUCUUUUCCGCCU	22		+				315	198	18.05	12.3924
miR-3049-5p	UCGGGAAGGCCGUUGCGGCGGA	22					+	503	245	28.8227	15.3341
miR-4510	UGAGGGAGUAGGUUGUAUUGUUU	23		+	+	+		381	323	21.8319	20.216
miR-iab-4	ACGUAUACUAAAUGUAUCCUGA	22	+	+	+	+	+	180	104	10.3143	6.5092
miR-iab-5p	UACGUAUACUAAAGGUAUACCGG	23	+		+	+	+	20	17	1.146	1.064

The abbreviations represent: aae, Aedes aegypti; ame, Apis mellifera; bmo, Bombyx mori; dme, Drosophila melanogaster; tca, Tribolium castaneum.

The plus symbols indicate: ++, miRNA sequences of B. tabaci were identical to those in other species; +, miRNA sequences of B. tabaci were similar to those in other species but differed in some nucleotide positions.

### Identification of Candidate Novel miRNA Candidates

In addition to the identification of conserved miRNAs, we identified 15 potential novel miRNA candidates in both B and Q libraries (Table 2). The length of the 15 predicted novel miRNA candidates ranged from 21 to 24 nt. The free energy of folding for these hairpin structures ranged from −32.22 kcal/mol to −18.6 kcal/mol. The read number for each novel miRNA was lower than that of the conserved miRNAs, which was consistent with previous studies. To investigate the conservation of these novel miRNA candidates in a wide range of animal species, we used these miRNAs as query sequences to perform BLASTn searches against all nucleotide sequences in miRBase v17.0 databases. No homologs were found in any animal species, suggesting that these newly identified miRNAs are all whitefly-specific.

**Table 2:** Novel miRNA candidates identified from *B. tabaci*.

Name	Sequence (5′–3′)	Length (nt)	Folding energy (kcal/mol)	Reads	
				B	Q
bta-miRn1	UAGGUGAAGCCGAAGAACUCGG	22	−27.60	7	3
bta-miRn2	UAUCAAUAGACAAAGGCUCGUAGC	24	−20.80	5	1
bta-miRn3	UGAACGGAUUUUGGGUUGGAGAGA	24	−30.10	26	0
bta-miRn4	UGCAAAGGUGCCGAAUGGACAGGG	24	−28.70	10	3
bta-miRn5	GAUGGACGAGGAGGAUCUGAC	21	−21.10	10	0
bta-miRn6	UUGGCCAUCCUGACACCCCUUG	22	−29.70	71	68
bta-miRn7	AUCGGUCUGGAUGACGUGACGC	22	−32.22	17	5
bta-miRn8	UAUGCGUCGCACAUGUAGUCGGC	23	−25.50	8	0
bta-miRn9	UGUAGAUUUUGGAGUGUCAAUGGA	24	−19.40	9	1
bta-miRn10	UCAGCCGUCGAAGAAGUCACUGA	23	−24.70	5	11
bta-miRn11	UUCGAAGAAUUUUUCCAUCUCU	22	−21.40	0	13
bta-miRn12	UAAGAGAUUUUCCGGACCAGCACU	24	−18.60	0	9
bta-miRn13	UACACGAUUGAUCUGGCAAGGCU	23	−24.40	1	6
bta-miRn14	UGAGUAUGAGAAUUGGACGCAGU	23	−19.10	3	6
bta-miRn15	UCUGAAACAGAGAGGGAUAUCGUG	24	−22.70	9	38

### Comparing the Expression of miRNAs between B and Q

**Figure 3:**
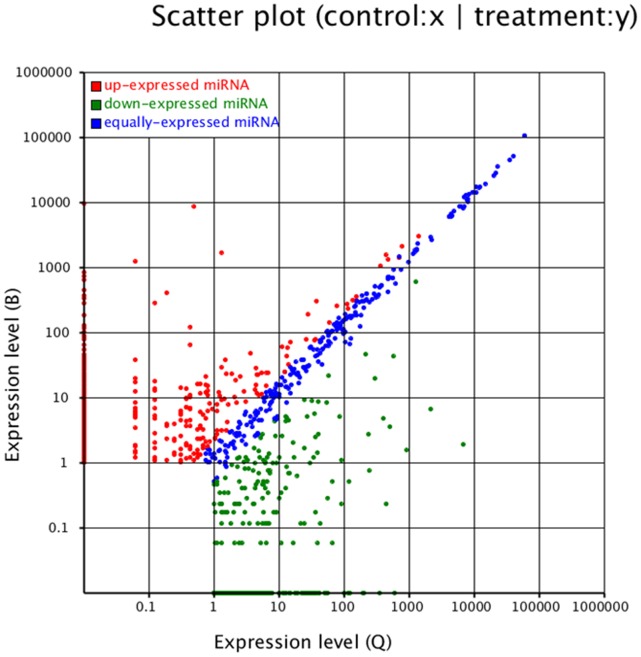
Expression of miRNAs in B vs. Q. After the expression of miRNAs was normalized, the expression levels (fold-change values = log_2_[B/Q]) of B were plotted against those of Q. Q was considered as the control and B was considered as the treatment.

To explore their difference in miRNAs, we compared the expression of miRNAs between B and Q (Fig. 3 and Table S1). The expression of 342 miRNAs was higher in the Q library than in the B library, and 198 miRNAs were found only in the Q library. The expression of 474 miRNAs was lower in the Q library than in the B library, and 303 miRNAs were found only in the B library. About 579 miRNAs were found in both B and Q libraries; among these, the expression of 144 miRNAs was higher in the Q library than in the B library, and the expression of 171 miRNAs was lower in the Q library than in the B library.

## Discussion

miRNAs post-transcriptionally regulate gene expression by targeting the 3′ untranslated region of specific messenger RNAs and causing mRNA degradation or translational repression [77], [78]. In this study, the expression of the most conserved miRNAs was 1.1 to 2.5 times greater in *B. tabaci* B than in Q (Table 1). Because miRNAs could recognize the target mRNAs based on sequence complementarity and cause mRNA degradation, the expression of some functional proteins should be lower in *B. tabaci* B than in Q, which possibly contributing to the biological differences between B and Q.

We found substantial differences between *B. tabaci* B and Q in the expression of miRNAs. For example, miR-139*, miR-1468, miR-4496, miR-1566, and miR-2993 were found only in *B. tabaci* Q, while miR-2687, miR-989b, miR-3178, miR-615, and miR-3070a were found only in B. The expression levels of these 10 miRNAs were very high, especially for miR-2687, which had 168,454 counts in B. In the 1080 miRNAs listed in the Table S1, only 268 miRNAs had similar normalized expression levels in B and Q libraries. Among the miRNAs, about 75.2% had substantially different expression levels in *B. tabaci* Q vs. B. Differences in the expression levels of these miRNAs could influence development, reproduction, insecticide resistance, apoptosis, etc., which might contribute to the displacement of B by Q.

*B. tabaci* Q has shown greater resistance to neonicotinoid and pyriproxyfen insecticides than B [47]–[49]. Accumulating evidence indicates an important role of miRNAs in drug resistance, and miRNA expression profiling is correlated with the development of drug resistance [79]–[81]. Although many studies have reported the involvement of miRNAs in drug resistance, few of these have concerned insects. We therefore analyzed the miRNAs possibly related to drug resistance on the basis of the studies in humans. In the present study, the normalized expression of miR-638 was 19,471 times higher in *B. tabaci* B than in Q (Table S1), suggesting a much lower level of drug resistance in B than in Q. Haenisch *et al.* [82] found that miR-379 increased (maximally 4.10±1.33-fold) in HepG2 cells after 48 h of treatment with 5 µM rifampicin. In our study, the normalized expression of miR-379 was 58 times higher in *B. tabaci* Q than in B (Table S1), again indicating a much greater drug resistance. Additional research is needed to determine whether miRNAs are involved in insecticide resistance.

This miRNA of miR-146b, which is highly homologous to miR-146a, was much more abundant in *B. tabaci* Q than B, and miR-146c was found only in Q (Table S1). The normalized expression of miR-215 was lower in *B. tabaci* Q than in B (Table S1), indicating lower apoptosis in Q. Apoptosis is considered a vital component of various processes including normal cell turnover, embryonic development, and chemical-induced cell death [83]. To date, several miRNAs have been identified that regulate the complex networks of apoptotic pathways [84]. Experimental evidence in human has demonstrated that miR-146a modulates activation-induced cell death (AICD), acting as an anti-apoptotic factor, and that was associated death domain (FADD) is a target of miR-146a [85]. Differences in the expression of these miRNAs might also explain difference in survival among *B. tabaci* biotypes. The tumor suppressor p53 acts as a major defense against cancer and can elicit both apoptotic death and cell cycle arrest [86]. miR-215 was identified as a p53-regulated miRNA [84], and induced cell cycle arrest [55], [87]. miR-215 which had pro-apoptotic function was detected at high levels in normal human colon tissue but at low levels in many human colon cancer samples. Once again, differences in expression of this miRNA in *B. tabaci* Q and B might contribute to biological differences in developmental time, reproduction, and survival.

### Conclusions

High-throughput sequencing enabled the study of miRNAs in *B. tabaci*, which is an important pest worldwide. We identified 170 conserved miRNAs and 15 novel miRNA candidates in B and Q. We found significant differences in the expression of miRNAs between B and Q, which might contribute to the displacement of B by Q. To date, little is known about the functions of these miRNAs in insects, especially in *B. tabaci.* Further analysis of the expression and function of these miRNAs could increase our understanding of regulatory networks in the insect and lead to novel approaches to its control.

## Supporting Information

Table S1 **Differential expression analysis of miRNAs between** ***Bemisia tabaci*** **B and Q.** A list of the 1,080 miRNAs in *B. tabaci* B and Q that were compared.
(XLS)
